# miR-429 RNA therapy as generic strategy to protect against photoreceptor loss

**DOI:** 10.1016/j.omtn.2025.102477

**Published:** 2025-02-15

**Authors:** Jan Wijnholds

**Affiliations:** 1 Department of Ophthalmology, Leiden University Medical Center (LUMC), Albinusdreef 2, 2333 ZA Leiden, the Netherlands; 2 Netherlands Institute for Neuroscience, an Institute of the Royal Netherlands Academy of Arts and Sciences (KNAW), Meibergdreef 47, 1105 BA Amsterdam, the Netherlands

## Main text

Ophthalmic patients with mutations in one of the over 318 identified inherited retinal disease (IRD) genes need gene therapy (http://sph.uth.edu/RETNET/). Since the US Food and Drug Administration (FDA) approval in 2017 of Luxturna as the first retinal gene-replacement therapy, the vision community is as of February 2025 still awaiting the next approved gene therapy product. Mutation-independent or generic therapies for patients with IRDs are therefore in high demand. A recent study published in *Molecular Therapy Nucleic Acids* by Petrogiannakis et al.^1^ reported a promising therapeutic strategy for the protection of photoreceptor cells (PRs) against cell death via overexpression of a precursor microRNA (miRNA) called pre-miR-429. Petrogiannakis and colleagues performed an *in vitro* high-content imaging (HCI) high-throughput screening assay in light-exposed mouse 661W cells and systematically scanned 1,268 miRNAs for miRNAs that protect against light-induced cell degeneration. The authors showed by subretinal delivery of recombinant adeno-associated-virus serotype 8 vector (AAV8.CMV.miR-429) protection against PR degeneration, preservation of electrophysiological responses, and reduced retinal inflammatory processes in the autosomal dominant *Rho*^P23H/+^ knockin retinitis pigmentosa (RP) mouse model.

IRDs such as non-syndromic RP, Leber congenital amaurosis (LCA), cone dystrophies, cone-rod dystrophies, macular dystrophies, and syndromic Usher and Bardet-Biedl disease occur, with an incidence of 1 in 3,500. For many of the IRD genes, there are hundreds of disease-causing mutation variations, with only a few mutation hotspots within the gene. Pathogenic mutations in essential genes in PRs, the retinal pigment epithelium (RPE), or Müller glial cells result in the loss of PRs. It will take a huge effort to develop the thousands of gene-specific clinical gene therapies, such as gene-augmentation, gene-editing, or antisense oligonucleotides (ASOs), for all the IRD genes and their manifold mutation variants. Of interest is that degeneration of PRs is in general due to common pathways such as mitochondrial oxidative stress, endoplasmic reticulum (ER) stress responses, inflammation, Ca^2+^ imbalance, high levels of cyclic guanosine monophosphate, deregulation of glycolysis, or activation of the innate and adaptive immune system. Previous studies by other scientists showed a slowdown in PR degeneration in different IRD mouse models after AAV vector-mediated gene augmentation of the truncated form of rod-derived cone viability factor (RdCVF, SPVN06) and the full-length form RdCVFL,^2^^,^^3^ or CRISPR-Cas gene editing of the prolyl hydroxylase domain-containing protein 2 gene (*PHD2*).^4^ These therapies affect in different ways the PR glycolysis in mutant mouse models of RP, but none of these generic therapies received European Medicines Agency or FDA approval, but ongoing natural history studies and encouraging clinical safety data have been reported in oral presentations at scientific meetings for SPVN06.

miRNAs are naturally occurring short RNA transcripts of 18–24 nt that regulate gene expression. miRNAs are transcribed from the genome into primary miRNAs and processed into precursor miRNA and miRNAs. miRNAs play essential roles in regulating the expression of key target messenger RNAs by binding to the 3′ untranslated region (3′ UTR), or less frequently to the 5′ UTR, coding region, or regulatory sequences of target genes. The authors of the recent study^1^ previously investigated the therapeutic potential of miR-204 in IRDs.^5^ They subretinally injected an AAV vector carrying the miR-204 precursor (AAV-miR-2024) in the RP *Rho*^P347S/+^ and LCA *Aipl1* IRD mouse models. The treated RP model showed preservation of the retinal function, reduced numbers of apoptotic PRs, better preservation of PR marker expression, and downregulation of microglia activation in the diseased retina. Protective effects were also observed in the treated LCA model. Other studies demonstrated that inhibition of miR-6937 by subretinal delivery of AAV-Anc80-inhibitor-miR-6937-5p in the *Pde6b*^rd10/rd10^ mouse model delays PR loss and increased the electroretinogram (ERG) response.^6^ Downregulation of miR-181a/b by subretinal administration of AAV8-inhibitor-miR-181a/b improved retinal morphology and increased the ERG response in *Rho*^P347S/+^ and *Pde6b*^rd10/rd10^ mouse models,^7^ suggesting that miRNAs are potential candidates to prevent progression of PR degeneration, but which miRNAs are the most potent ones in the mouse and human retina?

Using a systematic HCI screening assay on light-induced cultured mouse 661W cells, Petrogiannakis and colleagues^1^ identified six other miRNAs that after transfection conferred protection from light-induced damage. The miR-200 family member miR-429 was the top-scoring miRNA in *in vitro* tests, and the authors demonstrated protection of the mouse *Rho*^P23H/+^ retina without additional light exposure. *Rho*^P23H/+^ is a common model for fast-progressing RP with mutant rhodopsin misfolding in the ER. The mice show increased levels of intracellular calcium and activation of calpains as a mechanism of PR death.^8^ The five other cell-protective miRNAs are still to be tested, as are miRNAs from the HCI screen that stimulated PR cell death. In the limited number of mice tested, there was no significant improvement in PR outer nuclear layer thickness, but a strong, increasingly improved a-wave and b-wave ERG response was observed at postnatal day 30 (PN30), PN60, and PN110 in *Rho*^P23H/+^ retinas administered with AAV-miR-429 at PN8, a time point at which no pathological retinal phenotype is observed. In accordance with the enriched Gene Ontology biological processes obtained from the bulk RNA sequencing, the improved ERG response suggests that miR-429 applied at PN8 significantly improved the phototransduction cascade in the declining number of mutant PRs. When administered at PN20, a lesser therapeutic response is observed at PN60 and PN110, a time point at which the PR degeneration process peaks. It will be of interest to examine the window of miR-429 administration in between PN8 and PN20. The data suggest that AAV-miR-429 might be effective at clinically relevant disease stages. Long-lasting effects and clinical relevance of miR-429 need to be demonstrated in efficacy and safety studies in additional wild-type and fast and slow IRD models, small and large IRD animal models, and in human models such as wild-type cultured retinal explants and IRD retinal organoids. But will miR-429 work in human PRs? Currently, the mechanism of action of recombinant miR-429 is not known. The protective function by miR-429 on PRs might be mouse specific, but it is likely that miR-429 function is evolutionarily conserved. The authors used subretinal administration of AAV8.CMV.miR-429 containing a ubiquitous CMV promoter, which targets and expresses in PRs and RPE cells; therefore, indirect toxic levels of AAV-vector DNA and protective levels of miR-429 expression in RPE cells might play a role.^9^ Recombinant miR-429 may also be abundantly produced in RPE or RP cells, excreted, and effectively taken up by adjacent PR. Widespread target-cell to adjacent-cell biodistribution has been demonstrated, for example, upon intrastriatal miRNAs-based gene therapy for Huntington disease.^10^ Petrogiannakis and colleagues^1^ performed transcriptomics on miR-429-treated compared to nontreated *Rho*^P23H/+^ retinas and observed a downregulation of Gene Ontology terms related to innate immune response and inflammation, suggesting a relationship between microglia activation and neurodegenerative disease progression, which is another potential path for therapeutic applications.

The authors have added miR-429 to a short but urgent list of miRNA-based gene therapy strategies for IRDs. In the absence of gene-specific therapies, generic approaches such as downregulation of *PHD2* by AAV gene editing, AAV gene augmentation of RdCVF of RdCVFL, and the results presented by Petrogiannakis et al. on AAV miRNA augmentation of miR-429 in PR are highly encouraging and may present promising routes for clinical applications to prevent PR loss in IRDs.

## Acknowledgments

The author is funded by the European Commission grant HORIZON-MSCA-2021-DN-01.

## Declaration of interests

The author declares no competing interests.
